# Immunoactive Tripeptide RMK Alters T-Cell Subset Proportions and Remodels the Gut Microbiome–Metabolite Axis in S180 Tumor-Bearing Mice

**DOI:** 10.3390/nu18213628

**Published:** 2026-10-31

**Authors:** Rong Han, Qiuning Yang, Zhenjia Sun, Decheng Mao, Wenjie Ding, Yuemeng Wang, Haiyu Ji, Tian Wang

**Affiliations:** 1 Key Laboratory of Molecular Pharmacology and Drug Evaluation, Ministry of Education, Collaborative Innovation Center for Nutrition, Health, and Ecological Security of Food Resources in the Yellow River Basin, Advanced Drug Delivery System and Biotech Drugs in Universities of Shandong, School of Pharmacy, Yantai University, Yantai 264005, China; 19834286635@163.com (R.H.); y18037607620freya@163.com (Q.Y.); szj000925@163.com (Z.S.); decheng0313@163.com (D.M.); 2 College of Food Science and Engineering, Tianjin University of Science and Technology, Tianjin 300457, China; dingwenjie1120@163.com; 3 School of Food and Bioengineering, Yantai Institute of Technology, Yantai 264003, China; wangym0523@163.com

**Keywords:** immunoactive peptide, RMK, T-cell subsets, gut microbiome–metabolite axis, molecular docking

## Abstract

Background: Immunoactive peptides are emerging as safe oral immunomodulators, but whether a single tripeptide can alter T-cell subsets and the gut microbiome–metabolite axis is unclear. Methods: The synthetic tripeptide RMK (Arg–Met–Lys; HPLC purity > 98%) was given orally once daily to S180 sarcoma-bearing Kunming mice from 7 days before to 14 days after tumor inoculation, a host-preconditioning plus continuous-exposure regimen. Results: RMK reduced tumor growth at both doses (inhibitory rates 40.03% and 48.58%), attenuated thymic atrophy and splenomegaly, and was accompanied by higher proportions of thymic CD4^+^CD8^+^ double-positive cells, splenic CD4^+^ and CD8^+^ T cells and tumor-infiltrating CD8^+^ T cells. Tumor-derived suspensions showed mitochondrial depolarization and ROS accumulation, readouts of mitochondrial and redox status that do not identify a cell-death pathway. 16S rRNA sequencing showed higher relative abundances of Lachnospiraceae and Ruminococcaceae, although community structure did not differ significantly. Untargeted metabolomics showed altered bile acid, sphingolipid and steroid-hormone metabolites, with lower aldosterone and cortisol than in tumor-bearing controls. Molecular docking, as an exploratory prediction only, gave the most favorable poses for MHC-I/II (H-2Kb, ΔG = −7.09 kcal/mol) and TCR-proximal kinases (AKT1, ZAP-70, Lck). Conclusions: These findings identify RMK as a candidate oral immunomodulatory peptide whose site of action remains to be defined.

## 1. Introduction

Bioactive peptides—short sequences encrypted within proteins and released during enzymatic hydrolysis, fermentation or gastrointestinal digestion—are increasingly investigated as orally active modulators of host physiology [1,2]. Compared with small-molecule drugs they combine low intrinsic toxicity with high structural diversity and target selectivity and, in contrast to intact proteins, di- and tripeptides, can cross the intestinal epithelium largely intact through the proton-coupled oligopeptide transporter PepT1, which accepts essentially all possible di- and tripeptides [3,4]. PepT1-mediated uptake alone, however, does not establish that an intact tripeptide reaches the systemic circulation at concentrations sufficient to engage intracellular or cell-surface receptors, and this caveat applies to the present study. These features make short bioactive peptides credible candidates for oral immunomodulation.

The biological activity of a short peptide is dictated principally by its amino acid composition, and the basic residues arginine and lysine are among the most consequential determinants [5]. Their side chains remain protonated at physiological pH, so peptides enriched in Arg and Lys carry a net positive charge that favors electrostatic engagement of anionic membrane surfaces and of electronegative regions on cell-surface and intracellular receptors [6]. Arginine is distinctive because its guanidinium group supports bidentate hydrogen bonding, and arginine-rich sequences are among the most efficient known mediators of membrane translocation and cellular uptake [7]. Together with amphipathicity, this cationicity explains the sustained interest in repurposing cationic host-defense peptides as anticancer agents, with selectivity arising from the higher net negative surface charge and depolarized membrane potential of malignant cells [8]. Therefore, peptides enriched in basic residues may preferentially engage immune activation rather than exert indiscriminate cytotoxicity.

Cancer continues to rank among the primary causes of mortality globally, with roughly 20 million newly diagnosed patients each year [9], and malignant progression reflects not only cell-intrinsic alterations but also failure of immune surveillance [10]. CD8^+^ cytotoxic T lymphocytes are the decisive effectors in most settings: their density, spatial organization and differentiation state within the tumor predict clinical outcome more consistently than almost any other biomarker, and dysfunction or exhaustion of the intratumoral CD8^+^ compartment remains the principal obstacle to effective immunotherapy [11]. The supply of competent T cells depends on thymic output. Thymocytes progress through CD4^−^CD8^−^ double-negative, CD4^+^CD8^+^ double-positive (DP) and mature single-positive stages before emigrating to peripheral lymphoid organs [12]. Progressive tumor growth produces thymic involution characterized by selective depletion of DP thymocytes [13], so that restoring T-cell immunity is now a central therapeutic goal.

The gut microbiota has been established as a physiological rheostat of systemic immunity and an independent determinant of immunotherapy outcome: intestinal microbial composition distinguishes patients who respond to checkpoint blockade from those who do not, and transfer of fecal material transfers the phenotype [14,15]. Much of this influence is metabolic. Microbiota-derived short-chain fatty acids reinforce CD8^+^ T-cell effector and memory differentiation and improve the performance of both checkpoint blockade and adoptive cell therapy [16]. The relationship is bidirectional because tumors themselves distort gut microbial ecology and the resulting dysbiosis can reinforce systemic immune suppression, which makes the gut–metabolite–immune axis an attractive interventional node.

**Figure 1:**
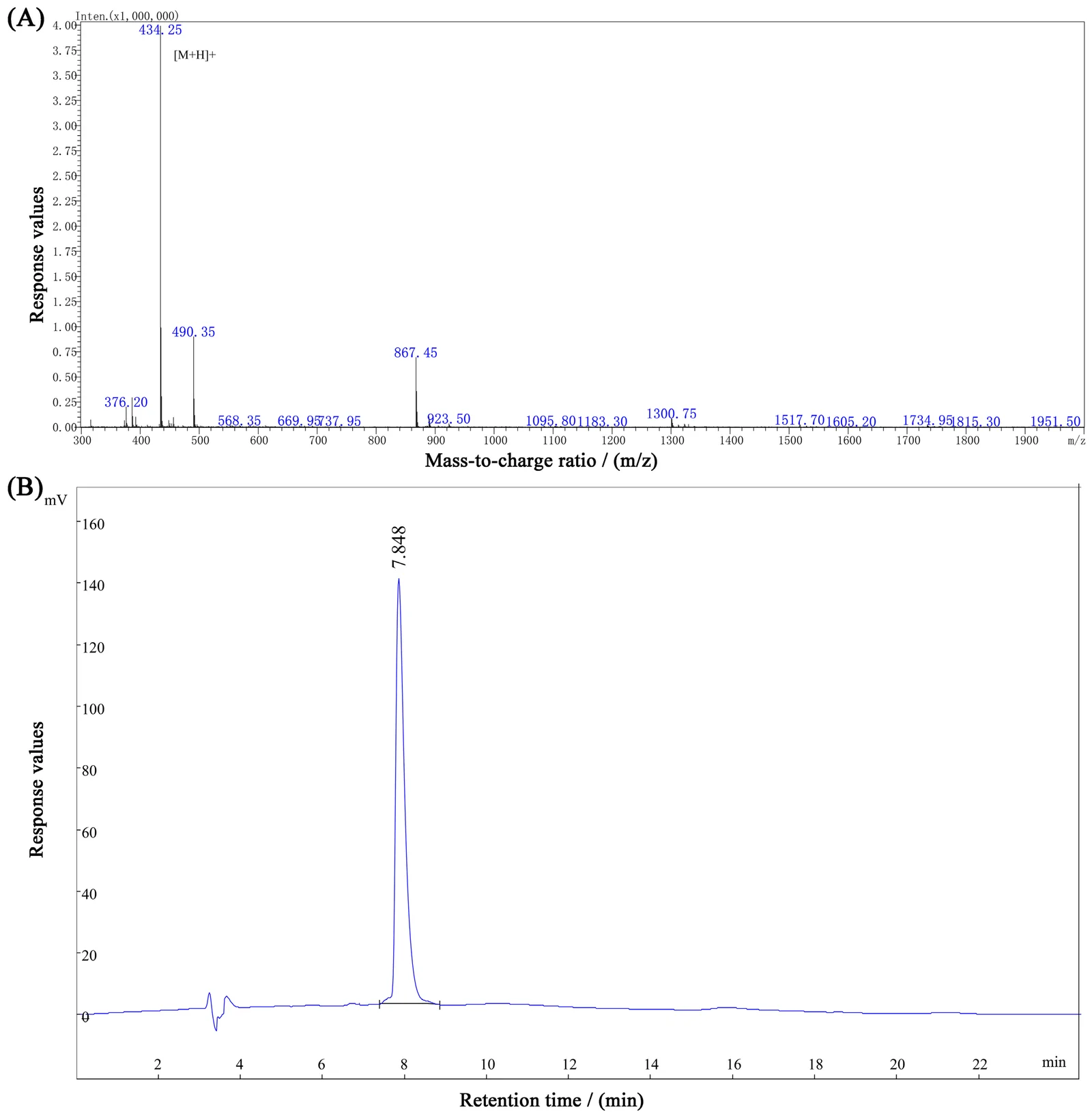
Identification of the synthetic tripeptide RMK. (**A**) Positive-ion ESI-MS spectrum showing the [M+H]^+^ ion at *m*/*z* 434.25. (**B**) RP-HPLC chromatogram showing a single symmetric peak at a retention time of 7.848 min.

RMK is a tripeptide composed of arginine, methionine and lysine (theoretical molecular mass 433.56 Da). The peptide was not selected at random or from computational prediction; it emerged from our own work on natural products with antitumor immunostimulatory activity, in which we compared the short-peptide profiles of intestinal contents from tumor-bearing mice [17,18]. In that comparison the intestinal level of RMK was almost abolished by tumor burden and rose again in mice receiving polysaccharide- or polyphenol-rich extracts that also increased Lactobacillus abundance and lymphocyte viability; these findings are unpublished preliminary observations and are therefore described only qualitatively. That observation prompted the synthesis of RMK at high purity (Section 2.1, Figure 1) and, in the present study, its direct oral administration as a single defined molecule. As outlined above, the Arg–Met–Lys composition provided a further, literature-based rationale because the RK motif carries a net positive charge at physiological pH and cationic Arg/Lys-rich sequences favor electrostatic engagement of anionic membrane surfaces and immunologically relevant receptors rather than indiscriminate cytotoxicity. The present work therefore differs from our earlier reports in three respects: RMK was administered as a chemically synthesized single peptide rather than being inferred from dietary intervention; its effects on T-cell subsets, the gut microbiota and the fecal and serum metabolome were assessed within one study; and candidate molecular targets were examined by docking as explicitly exploratory predictions. Accordingly, flow cytometry, 16S rRNA gene sequencing, untargeted fecal and serum metabolomics, and molecular docking were integrated to investigate the effects of orally administered RMK on T-cell subsets and gut microbiota-associated metabolism in tumor-bearing mice. The underlying mechanisms were explored at four levels—immune phenotype, microbial community structure, host metabolism, and candidate molecular targets. These results provide a basis for the further evaluation of RMK as an oral immunomodulatory peptide in the food and pharmaceutical fields.

The study should be read within explicit bounds. First, it is an exploratory investigation of host preconditioning followed by continuous peri-inoculation exposure, and it does not address the treatment of manifest disease. Second, the four levels of analysis—immune phenotype, microbial community structure, host metabolism and candidate molecular targets—are reported as parallel readouts rather than as links in a demonstrated causal chain; the immunological, microbial and metabolic observations are associations, and their order in this article reflects the sequence of the assays rather than a proposed mechanism. Third, the docking results are predictions generated in silico and are not measurements of binding, target engagement or pathway activation. For these reasons the study does not identify the tissue compartment in which an orally administered tripeptide acts, and the pharmacological exposure of intact RMK is not defined here.

## 2. Materials and Methods

### 2.1. Reagents and Antibodies

Synthetic RMK (purity ≥ 98%, lot NJP92758-240607) was obtained by Fmoc solid-phase peptide synthesis from Nanjing Peptide Biological Technology Co., Ltd. (Nanjing, China). PE-conjugated CD4 and FITC-conjugated CD8 antibodies were purchased from ImmunoWay Biotechnology (Plano, TX, USA). Rhodamine 123 (Rh123) and 2′,7′-dichlorodihydrofluorescein diacetate (DCFH-DA) were from MedChemExpress (Monmouth Junction, NJ, USA).

### 2.2. Identification of RMK

The molecular weight was characterized using electrospray ionization mass spectrometry (ESI-MS). Measurements were performed on a Shimadzu LCMS-2020 single-quadrupole mass spectrometer (Shimadzu, Kyoto, Japan) under positive-ion mode. Purity was assessed by reversed-phase HPLC on a Shimadzu LC-20AD series system with UV detection (Shimadzu, Kyoto, Japan), using a ChromCore 120 C18 column (4.6 × 250 mm, 5 μm, NanoChrom, Suzhou, China) and a linear gradient of 0.1% trifluoroacetic acid in acetonitrile (10% to 35% A over 25 min at 1.0 mL/min) and 0.1% trifluoroacetic acid in water with detection at 220 nm and a 5 μL injection, and was calculated by peak-area normalization [19].

### 2.3. Experimental Design

Forty SPF male Kunming mice (8 weeks old, body weight 20–25 g) were maintained at 22–25 °C with 40–70% relative humidity and a 12 h light–dark cycle. Animals had ad libitum access to standard rodent chow and autoclaved water [20]. After 1 week of acclimatization, mice were allocated to 4 groups (10 mice per group, each group was housed in a separate cage) using a computer-generated randomization sequence: blank control (Blank), tumor model (Model), RMK low-dose (RMK-L, 25 mg/kg) and RMK high-dose (RMK-H, 50 mg/kg). The RMK dose was selected from preliminary tests, as excessively high or low doses could not establish a valid gradient. RMK-L and RMK-H mice received RMK by oral gavage once daily for 7 days before tumor inoculation (pre-feeding phase); blank and model mice received an equal volume of sterile saline on the same schedule. All mice except the blank group were then inoculated subcutaneously into the right axillary flank with S180 sarcoma cells (2 × 10^6^ cells/mouse), and dosing continued for a further 14 days. The regimen therefore combined 7 days of pre-inoculation dosing with 14 days of continuous post-inoculation dosing, i.e., host preconditioning plus continuous exposure. For subsequent experimental assays, five mice whose body weights were close to the average were selected. Three of these five mice were further randomly selected for representative gut microbiota profiling and untargeted metabolomic analysis. Blinding was implemented throughout the animal experiment. Experimental grouping, intragastric administration and data analysis were carried out by separate researchers. Because no group received RMK only after the tumor had become established, the design cannot separate an effect on tumor establishment from an effect on the growth of an already established tumor.

### 2.4. Organ Indices and Tumor Growth

At the pre-determined experimental endpoint (day 14 after tumor inoculation), mice were fasted for 12 h with free access to water, weighed, and euthanized by cervical dislocation. Death was confirmed by the absence of a heartbeat and of a corneal reflex before dissection. No adverse events, including mortality, morbidity were observed in any group at any point during the study. No humane endpoints were pre-defined for this study; accordingly, all animals were maintained until the scheduled experimental endpoint and were then euthanized as described above. Thymuses, spleens and tumors were excised, rinsed in ice-cold saline, blotted dry, and weighed on an analytical balance. Thymuses, spleens and tumors were excised, rinsed in ice-cold saline, blotted and weighed. Organ indices and tumor inhibitory rate were calculated as: organ indices (mg/g) = organ weights (mg)/body weights (g); tumor inhibitory rates (%) = (tumor weights of model group − tumor weights of treated groups)/tumor weights of model group × 100% [21].

### 2.5. Flow Cytometric Analysis of T-Cell Subsets

Single-cell suspensions were prepared from thymus, spleen, peripheral blood, and solid tumor; the mononuclear cells were isolated after erythrocyte lysis and then resuspended in staining buffer and incubated with PE-anti-CD4 and FITC-anti-CD8 antibodies for 30 min at 4 °C in the dark, washed twice, and acquired on a flow cytometer (Beckman Coulter, CytoFLEX, Indianapolis, IN, USA) following current guidelines for flow cytometric immunophenotyping [22]. Events were first gated on the lymphocyte region in a forward- versus side-scatter plot and then on singlets in a forward-scatter area versus height plot and finally displayed on a CD4 versus CD8 plot; compensation was applied using single-stain preparations. No CD3 co-staining and no live/dead viability dye were used, and no fluorescence-minus-one or counting-bead controls were included; the CD4^+^CD8^+^ events reported here therefore represent co-expression of the two markers within the lymphocyte gate rather than a CD3-confirmed double-positive population. Data were analyzed using CytExpert software (v2.5), and the results were expressed as the proportions of CD4^+^, CD8^+^ and CD4^+^CD8^+^ cells among gated events rather than as absolute cell numbers, with five mice analyzed per group.

### 2.6. Mitochondrial Membrane Potential and Intracellular ROS

Freshly prepared tumor-derived cell suspensions were divided into two aliquots and incubated at 37 °C in the dark with Rh123 (5 μmol/L, 30 min) to report mitochondrial membrane potential (Δ*Ψm*) or with DCFH-DA (10 μmol/L, 20 min) to report intracellular ROS. Rh123 accumulates in mitochondria in proportion to Δ*Ψm*, so loss of Rh123 fluorescence identifies cells with depolarized mitochondria; oxidation of DCFH-DA to fluorescent DCF is proportional to intracellular ROS. The tumor-derived suspensions were neither fractionated nor stained with a lineage or tumor-cell-specific marker, so the analyzed events comprise malignant cells together with infiltrating leukocytes and stromal cells; these readouts therefore describe all viable cells in the suspension rather than the malignant compartment. Samples were washed and analyzed immediately using CytExpert software (v2.5) of flow cytometry (Beckman Coulter, CytoFLEX, IN, USA) [23,24].

### 2.7. 16S rRNA Gene Sequencing

Total genomic DNA was isolated from fecal specimens. The V3–V4 hypervariable region of bacterial 16S rRNA gene was amplified with barcoded universal primer pairs (338F/806R), and three technical replicates were prepared for each sample. Purified amplicons were quantified via QuantiFluor™-ST (Promega, Madison, WI, USA), and libraries were constructed and sequenced on Illumina MiSeq (San Diego, CA, USA) with PE250 paired-end strategy. Three biological replicates per group were sequenced; each sample yielded 101,457–116,519 raw reads, of which 77.7–82.1% were retained as non-chimeric sequences. Quality trimming and merging of raw reads were performed using FASTP (v0.23.2) and FLASH (v1.2.11), respectively; high-quality clean reads were imported into QIIME 2 (v2023.9). Host mouse sequences were filtered by Kraken2 (v2.1.2), and DADA2 plugin (v2022.2) was applied for quality trimming, paired merging, denoising and chimera filtering to generate ASVs, which were taxonomically classified against the SILVA 138 database. Alpha diversity (Shannon index, Pielou evenness and Faith’s phylogenetic diversity) was compared among groups by the Kruskal–Wallis test, and beta diversity was assessed with the Jaccard distance and visualized by principal coordinates analysis; differences in overall community structure were tested by PERMANOVA (adonis). Counts were analyzed without compositional transformation, and because 16S rRNA data are compositional the reported shifts should be read as differences in relative abundance. LEfSe analysis was used to identify differential taxa between groups with a significance level of *p* < 0.05; the LDA score > 4.0, and no correction for multiple testing was applied, so these results are exploratory [25].

### 2.8. Untargeted Metabolomics

Fecal samples were homogenized and deproteinized with methanol, while serum samples were deproteinized using acetonitrile. Following centrifugation, the obtained supernatants were analyzed by ultra-performance liquid chromatography coupled with quadrupole time-of-flight mass spectrometry (UPLC-Q-TOF MS, Waters, Milford, MA, USA). Both positive and negative electrospray ionization modes were adopted for detection. Separation was performed on a C18 analytical column (2.1 mm × 100 mm, 1.7 μm) with gradient mobile phases, and MS source parameters were optimized for untargeted metabolomic profiling of biofluids. Raw UPLC–Q-TOF MS data were processed using Progenesis QI (Waters, Milford, MA, USA) for peak picking, alignment and normalization. Unsupervised principal component analysis (PCA) and supervised orthogonal partial least squares–discriminant analysis (OPLS-DA) were performed with SIMCA (Sartorius, Göttingen, Germany). Differential metabolites were screened on the criteria of VIP > 1.0 from the OPLS-DA model and *p* < 0.05 from a two-tailed Student’s *t*-test; no false-discovery-rate correction was applied to these comparisons, and three biological replicates per group were analyzed. Accurate mass screening and MS/MS spectrum comparison against public databases were applied for metabolite annotation in accordance with conventional untargeted metabolomics reporting standards; no authentic standards were analyzed in parallel, so all assignments are putative and correspond approximately to MSI confidence levels 2–3. Subsequently, KEGG pathway enrichment analysis was conducted based on the annotated metabolic features [26]. Complete metabolite lists were provided in Supplementary Tables S1–S4.

### 2.9. Molecular Docking

Crystal structures of 18 targets related to T-cell development, antigen presentation, TCR-proximal signaling, lineage-specifying transcription factors, co-stimulatory receptors and immune checkpoints were retrieved from the Protein Data Bank; seven murine and eleven human structures were used, the murine entries corresponding to molecules for which murine structures were available and the human entries to kinases and receptors for which only human structures are well resolved. Target receptors were processed in AutoDockTools (version 1.5.6): only the chain containing the functional domain was retained, water molecules, co-crystallized ligands, glycan chains and antibody, TCR or DNA chains were removed, hydrogens and Gasteiger charges were added with OpenBabel, and the structures were exported as PDBQT files. The RMK tripeptide was built with RDKit, 40 conformers were generated with ETKDGv3 and optimized with the MMFF94 force field, and the molecule was protonated as expected at physiological pH (net charge +2) and converted to PDBQT with Meeko. Molecular docking was performed with AutoDock Vina v1.2.5. Grid boxes of 22 × 22 × 22 Å to 28 × 28 × 28 Å were used according to the type of pocket, and the box center was placed on the functional pocket, defined by the centroid of the co-crystallized ligand, of the peptide chain or of the range of key residues. Exhaustiveness was set to 16, num_modes to 10, energy_range to 4 kcal/mol and the random seed to 42. For each target the pose with the lowest binding free energy (ΔG, kcal/mol) was selected, and residues within 4 Å of the ligand were identified. Docking conformations were visualized using PyMOL 2.4 (Schrödinger, New York, NY, USA) and Discovery Studio [27]. The docking protocol itself was not validated: no co-crystallized ligand was redocked, no decoy set was used to estimate enrichment, no alternative scoring function was applied for consensus ranking, no control peptide was included, and no biochemical validation of the predicted interactions was performed. A ΔG of −5.0 kcal/mol or below was used as an operational reporting threshold and not as a validated criterion of affinity, and scores obtained for structurally unrelated proteins are not comparable with one another. Because a tripeptide is substantially shorter than the 8–10-residue and 13–25-residue peptides accommodated by MHC class I and class II grooves, occupancy of a groove in these poses cannot be taken as evidence of altered antigen presentation; and because eleven of the receptors were human structures whereas the biological experiments were performed in mice, the computed scores cannot be used to explain the murine phenotype.

### 2.10. Statistical Analysis

All quantitative results are presented as the mean ± standard deviation (SD). Group differences were assessed by one-way analysis of variance (ANOVA) after verification of homogeneity of variance, and the least significant difference (LSD) procedure was applied only when the omnibus F test reached *p* < 0.05 (protected LSD). The pairwise comparisons were specified in advance, namely each group versus the blank group and each treated group versus the model group, so that the comparisons were fixed before analysis and the family-wise error rate was kept close to the nominal level; the *p* values are nevertheless reported without adjustment, and the analyses are regarded as exploratory. All statistical analyses complied with the standard biomedical experimental design and reporting specifications [28]. A *p* value less than 0.05 was defined as the threshold of statistical significance.

## 3. Results

### 3.1. Identification and Purity of Synthetic RMK

In positive-ion ESI-MS, RMK gave a prominent quasi-molecular ion [M+H]^+^ at *m*/*z* 434.25, consistent with the theoretical mass of the Arg–Met–Lys tripeptide (433.56 Da) (Figure 1A). Minor ions were attributable to adduct and dimer species, and no signals indicative of deletion sequences or protecting-group residues were detected. RP-HPLC resolved a single, sharp and symmetrical peak at a retention time of 7.848 min, with purity exceeding 98% by peak-area normalization (Figure 1B). Together these data establish the identity and suitability of the material used for the following in vivo experiments [19].

### 3.2. RMK Suppresses Tumor Growth and Normalizes Immune Organ Indices

**Figure 2:**
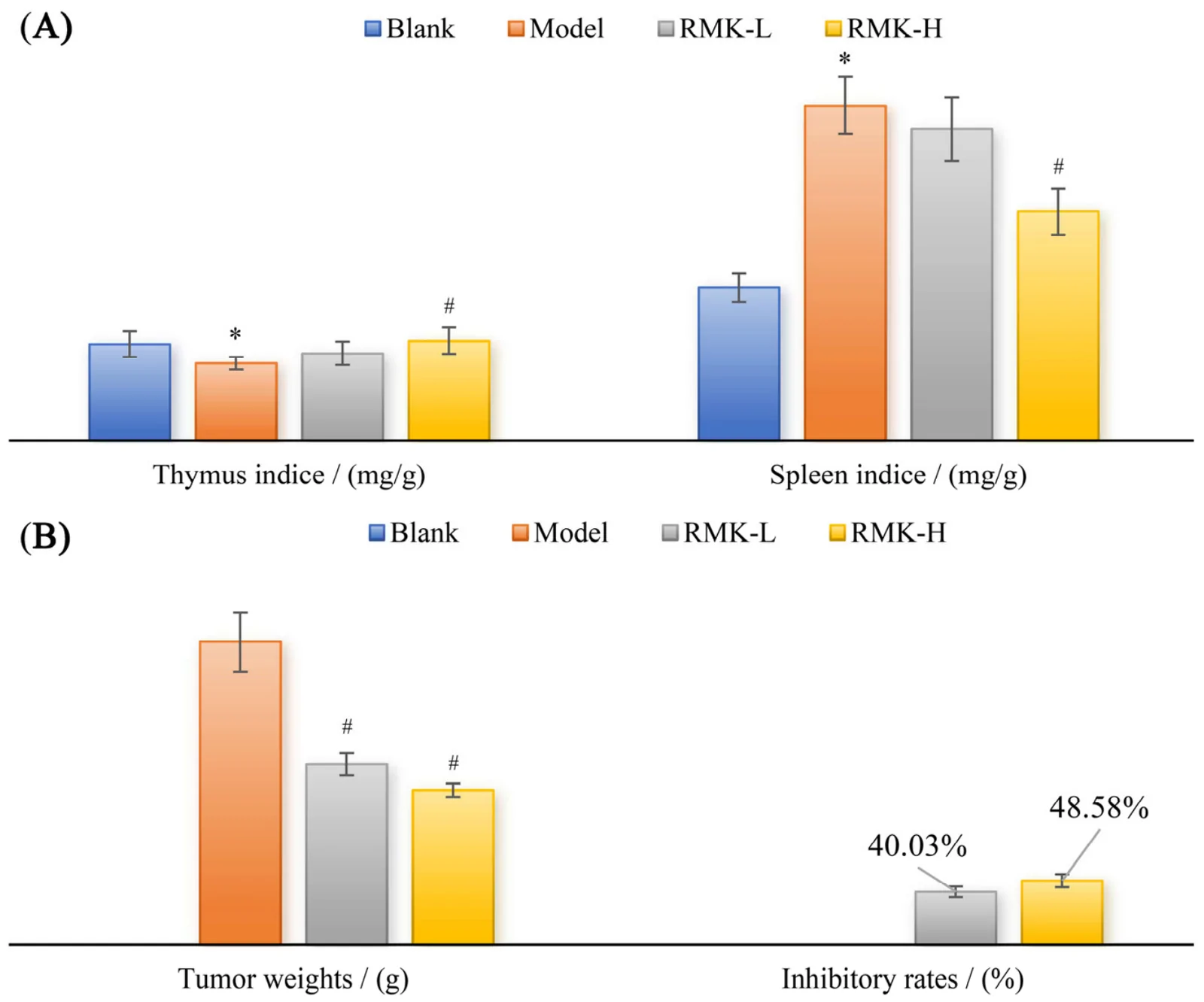
Effects of RMK on immune organ indices and tumor growth in tumor-bearing mice (*n* = 5). (**A**) Thymus and spleen indices. (**B**) Tumor weight and corresponding tumor inhibitory rates (40.03% for RMK-L; 48.58% for RMK-H). Note: * *p* < 0.05 versus blank; # *p* < 0.05 versus model.

Relative to the mice in the blank group, the model group showed a significant decrease in thymus indices and a significant increase in spleen indices (*p* < 0.05), the combination of central atrophy and peripheral enlargement that was characteristic of established tumor burden [13]. RMK administration attenuated these tumor-associated shifts rather than restoring them: thymus and spleen indices in the high-dose group approached, but did not reach, blank-group values (Figure 2A). Both RMK treatments significantly reduced tumor weights (*p* < 0.05) compared with the model group, yielding inhibitory rates of 40.03% (RMK-L) and 48.58% (RMK-H), with a numerically greater inhibition at the high dose (Figure 2B); the magnitude of inhibition was within the range reported for orally administered food-derived products [29].

### 3.3. RMK Alters T-Cell Proportions in the Thymus and Spleen

**Figure 3:**
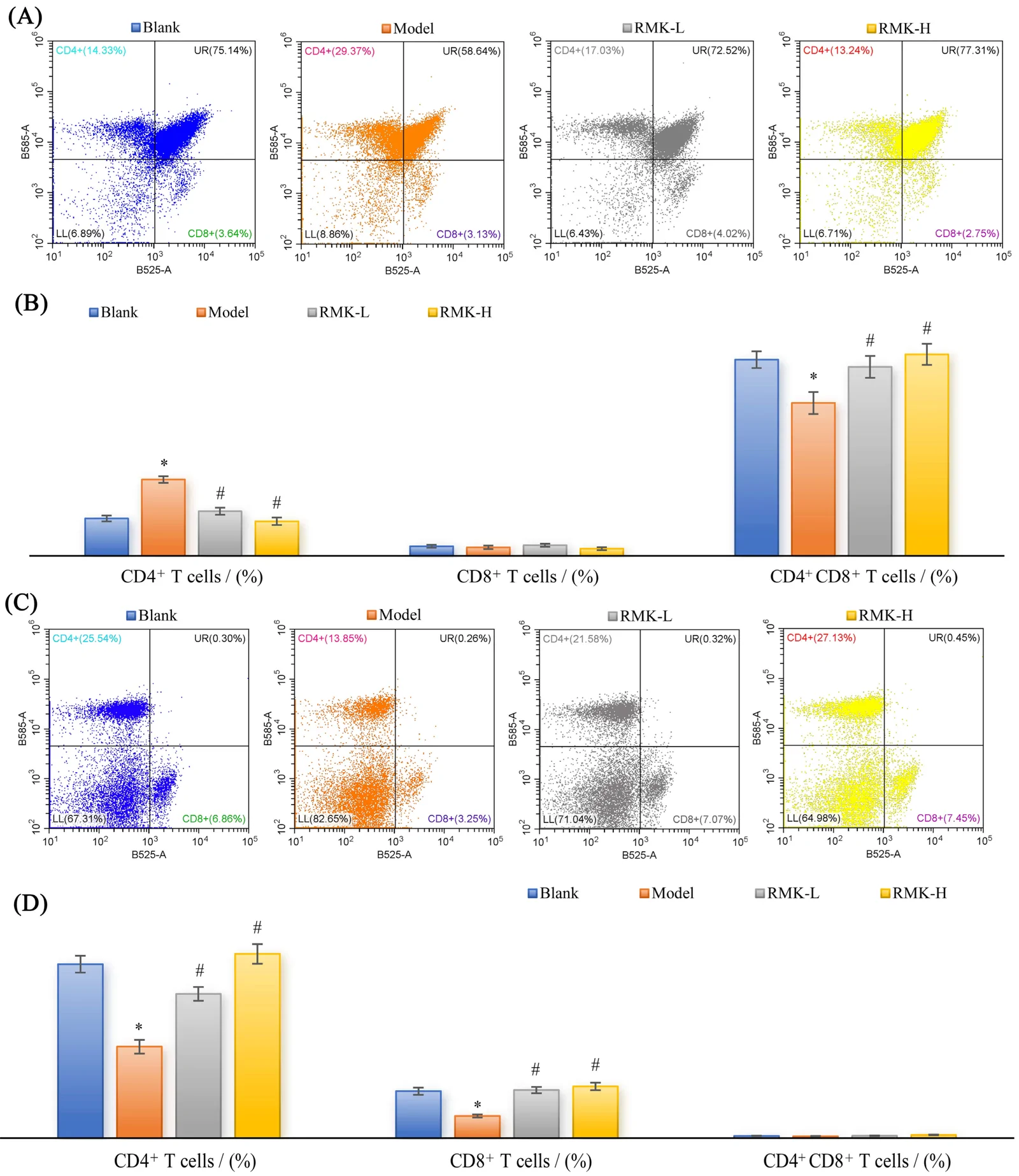
Effects of RMK on T-cell subsets in the thymus and spleen (*n* = 5). (**A**) Representative CD4/CD8 flow cytometry plots of thymocytes; (**B**) quantification of thymic CD4^+^, CD8^+^ and CD4^+^CD8^+^ T cells; (**C**) representative CD4/CD8 plots of splenocytes; (**D**) quantification of splenic T-cell subsets. Note: * *p* < 0.05 versus blank; # *p* < 0.05 versus model.

CD4^+^ T helper cells coordinate adaptive immune responses, whereas CD8^+^ cytotoxic T lymphocytes exert direct tumor-killing effects. Flow cytometric analysis of thymocytes revealed disrupted T-cell development in tumor-bearing mice (Figure 3A,B). Compared with the blank group, mice in the model group exhibited significantly reduced double-positive (DP, CD4^+^CD8^+^) thymocytes (*p* < 0.05) and significantly elevated CD4^+^ single-positive (SP) thymocytes (*p* < 0.05), a pattern consistent with tumor-induced perturbation of thymocyte differentiation. RMK treatment partially offset this shift, with a higher proportion of DP thymocytes and a decrease in CD4^+^ SP thymocytes to levels comparable to the blank group (*p* < 0.05). A higher proportion of DP thymocytes may also arise from contraction of other thymocyte subsets because total thymocyte numbers were not determined. In addition, tumor burden also induced obvious changes in splenic T-cell subsets (Figure 3C,D). The proportions of splenic CD4^+^ and CD8^+^ T cells were significantly decreased in the model group relative to those in the blank group (*p* < 0.05). RMK administration significantly increased the fractions of both splenic CD4^+^ and CD8^+^ T cells compared with the model group (*p* < 0.05), indicating higher splenic CD4^+^ and CD8^+^ proportions than in model mice [30]. All data in this section are expressed as percentages of cells within the respective gate; absolute cell numbers and total organ cell counts were not measured, so a higher percentage of any subset, including DP thymocytes, cannot be equated with increased thymic output.

### 3.4. RMK Alters Circulating and Tumor-Infiltrating T-Cell Subsets

**Figure 4:**
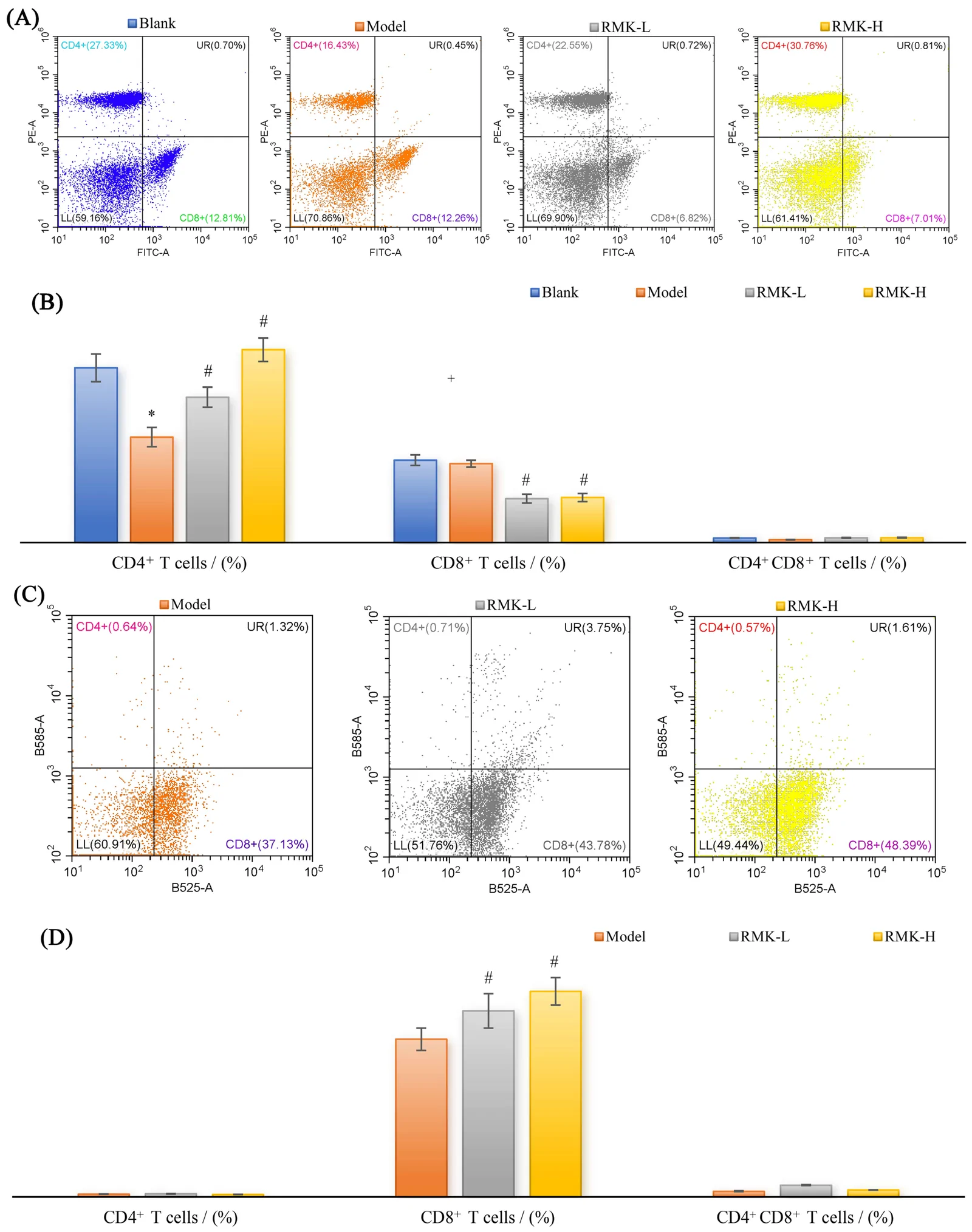
Effects of RMK on circulating and tumor-infiltrating T-cell subsets (*n* = 5). (**A**) Representative CD4/CD8 plots of peripheral blood lymphocytes. (**B**) Quantification of circulating T-cell subsets. (**C**) Representative CD4/CD8 plots of tumor-infiltrating lymphocytes. (**D**) Quantification of tumor-infiltrating T cells. Note: * *p* < 0.05 versus blank; # *p* < 0.05 versus model.

Flow cytometric analysis of peripheral blood lymphocytes revealed altered circulating T-cell profiles in tumor-bearing mice (Figure 4A,B). Compared with the blank group, the model group exhibited a significantly decreased proportion of circulating CD4^+^ T cells (*p* < 0.05), while CD8^+^ T cells showed no obvious change. RMK treatment significantly increased the percentage of peripheral CD4^+^ T cells in both RMK groups (*p* < 0.05). By contrast, circulating CD8^+^ T cells were markedly reduced after RMK intervention (*p* < 0.05). The percentages of CD4^+^CD8^+^ T cells remained negligible across all groups. Tumor-infiltrating lymphocytes (TILs) were further analyzed to evaluate the intratumoral immune microenvironment (Figure 4C,D). Compared with the model group, RMK administration significantly elevated the fractions of intratumoral CD8^+^ T cells in both RMK groups (*p* < 0.05). The proportions of tumor-infiltrating CD4^+^ T cells and CD4^+^CD8^+^ T cells remained at low levels, with no marked differences observed between the model group and RMK groups. All values in this section are proportions of gated cells; absolute numbers of circulating or intratumoral T cells and total tissue cellularity were not determined.

CD8^+^ T-cell density within the tumor is among the strongest correlates of outcome in cancer and a determinant of response to immunotherapy [31], and insufficient intratumoral T-cell infiltration is a well-established cause of immunotherapy failure [32]. In the present data, a lower proportion of circulating CD8^+^ T cells and a higher proportion of intratumoral CD8^+^ T cells were observed in the same animals. Because only relative proportions of gated cells were determined, and because no labeling, adoptive transfer or chemokine profiling was performed, this pattern is equally compatible with altered egress from the circulation, margination or redistribution between compartments, a change in tumor cellularity, or a change in the size of the other populations that make up the denominator within the gate. The present study cannot distinguish between these possibilities, and the two observations are therefore reported side by side rather than as evidence of recruitment or retention of CD8^+^ effector T cells within tumor tissue.

### 3.5. RMK Is Accompanied by Mitochondrial Depolarization and Oxidative Stress in Tumor-Derived Cell Suspensions

**Figure 5:**
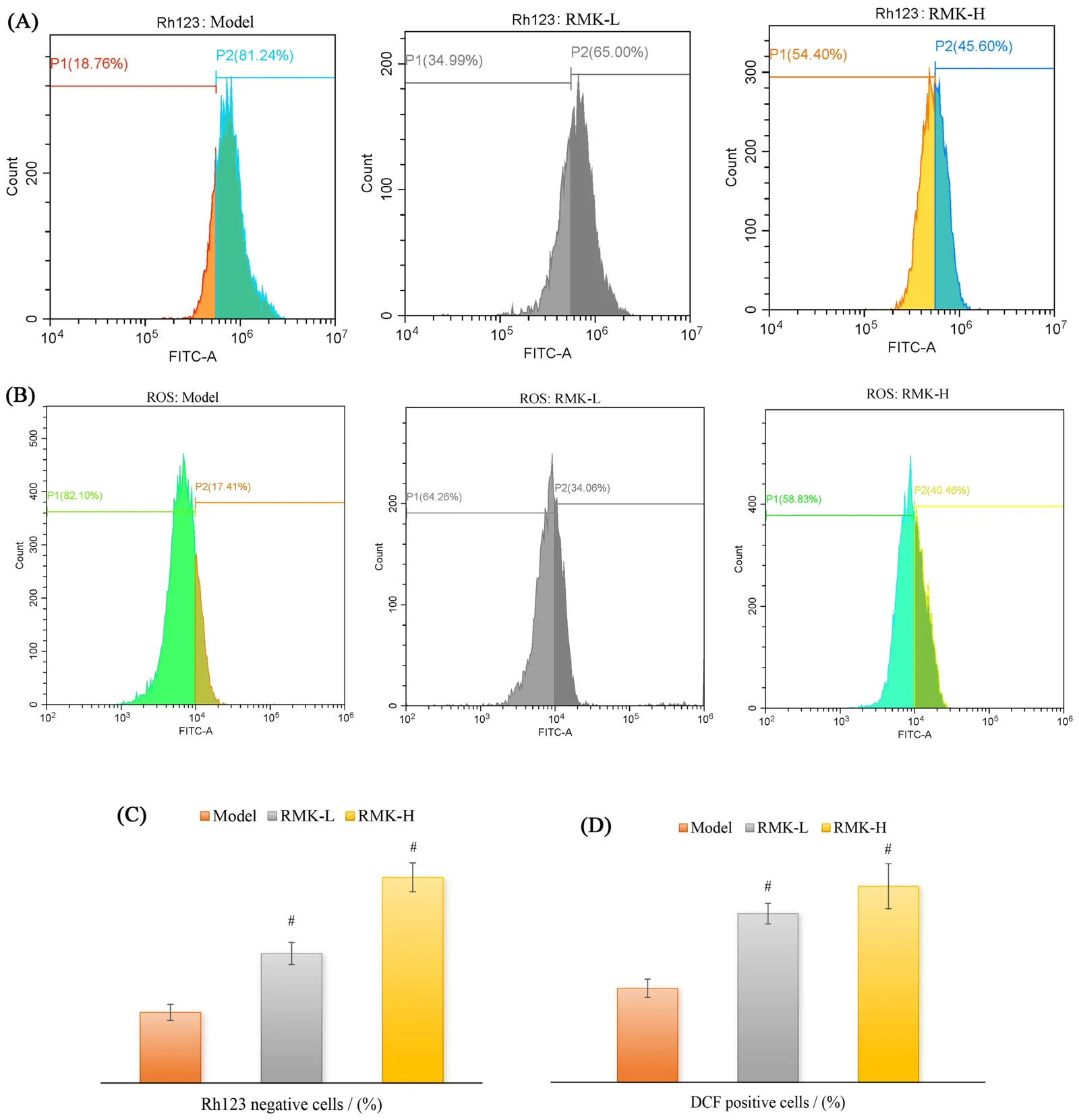
Effects of RMK on mitochondrial membrane potential and reactive oxygen species in unfractionated tumor-derived cell suspensions (*n* = 5). (**A**) Representative Rh123 histograms. (**B**) Representative DCF histograms. (**C**) Quantification of Rh123-negative (depolarized) events. (**D**) Quantification of DCF-positive (ROS-high) events. Note: # *p* < 0.05 versus model.

Mitochondrial membrane potential (MMP) and reactive oxygen species (ROS) are key biomarkers of mitochondrial and redox status. These assays were performed on unfractionated tumor-derived cell suspensions, and no lineage marker was used to identify malignant cells; the events analyzed therefore comprise malignant cells together with infiltrating leukocytes and stromal cells, and the readouts below describe the whole tumor-derived suspension rather than the tumor-cell compartment. Rh123, a fluorescent probe sensitive to membrane potential, was used to evaluate MMP; Rh123-negative events denote cells with depolarized mitochondria within this suspension (Figure 5A,C). Compared with the model group, RMK treatment significantly increased the proportions of Rh123-negative cells in both RMK groups (*p* < 0.05), indicating a higher proportion of cells with depolarized mitochondria. Intracellular ROS production was further measured via DCF fluorescence staining (Figure 5B,D). The percentage of DCF-positive (ROS-high) cells was markedly up-regulated following RMK administration in both RMK groups (*p* < 0.05). Loss of Δ*Ψm* together with ROS accumulation is a recognized index of mitochondrial stress [33], and ROS is also a recognized component of redox signaling [34]; both probes report mitochondrial and redox status only. Annexin V-FITC/PI staining and caspase-3/PARP or Bax/Bcl-2 measurement were not performed, so these observations indicate mitochondrial stress but do not identify a mode of cell death.

### 3.6. RMK Alters the Relative Abundance of Specific Gut Taxa

**Figure 6:**
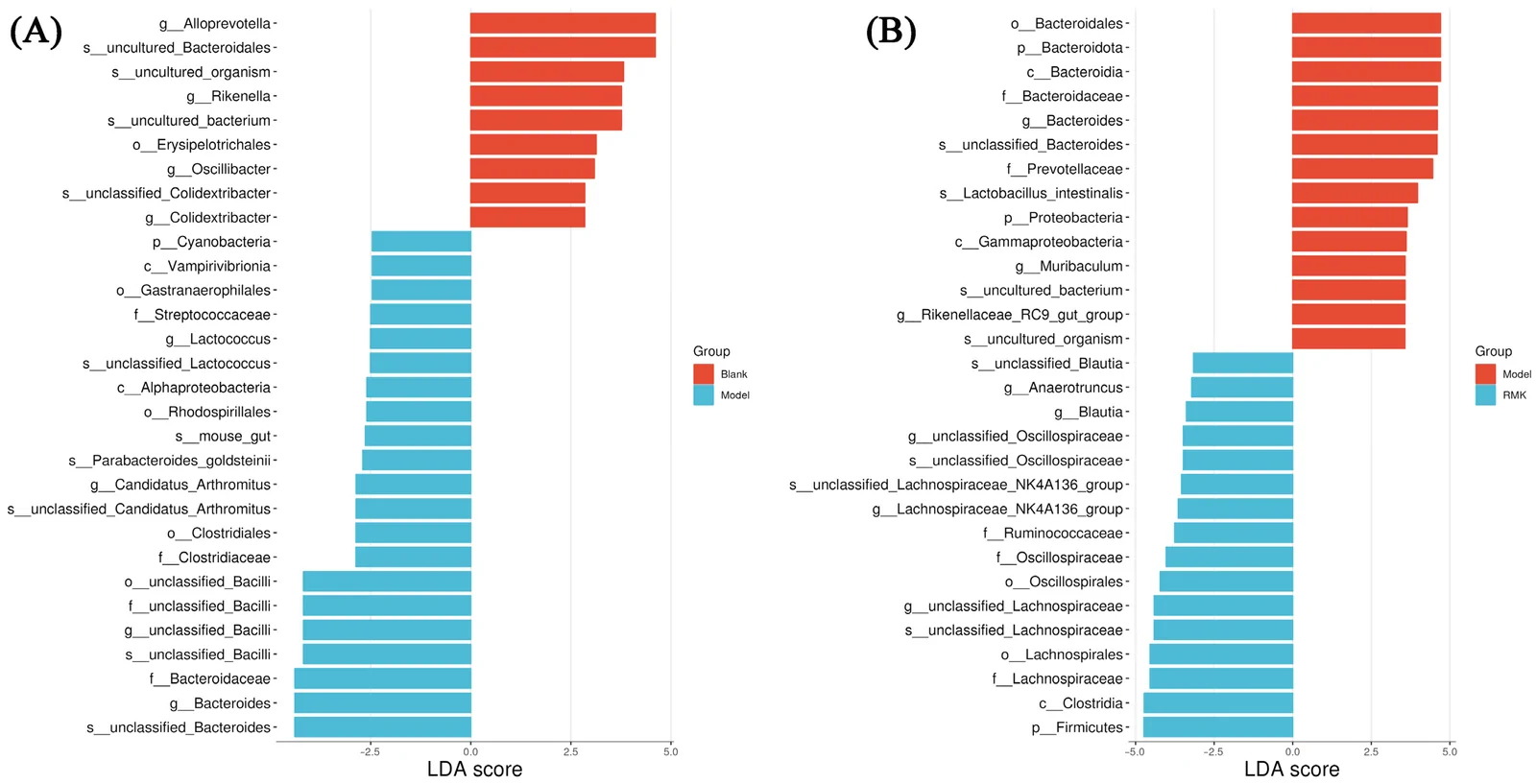
Differentially abundant bacterial taxa identified by LEfSe (LDA score > 4.0, *p* < 0.05, uncorrected for multiple testing; *n* = 3). (**A**) Differentially abundant taxa between blank and model groups. (**B**) Differentially abundant taxa between model and RMK groups.

Three biological replicates per group were sequenced, and community-level structure did not separate significantly between any two groups (Jaccard PERMANOVA: Blank vs. Model R^2^ = 0.171, *p* = 0.90; Model vs. RMK R^2^ = 0.216, *p* = 0.20; Blank vs. RMK R^2^ = 0.194, *p* = 0.50). Alpha diversity did not differ among the groups (Shannon index 5.72 ± 0.81, 5.76 ± 0.43 and 6.19 ± 0.41 for Blank, Model and RMK; Kruskal–Wallis H = 1.16, *p* = 0.561), and neither Pielou evenness (*p* = 0.875) nor Faith’s phylogenetic diversity (*p* = 0.430) separated the groups. The differences described below are therefore taxon-specific changes in relative abundance and do not constitute an ecosystem-level recovery. The LEfSe algorithm was used to screen for bacterial taxa exhibiting abundance differences among the experimental groups (Figure 6). Comparison between the blank and model groups identified distinct microbial biomarkers (Figure 6A). Taxa enriched in the blank group were mainly members of *Alloprevotella*, *Rikenella* and *Oscillibacter*, whereas the model group was characterized by enrichment of *Bacteroides*, *Lactococcus* and other taxa. The LEfSe comparison between the model and RMK groups further uncovered abundance shifts associated with RMK treatment (Figure 6B). Multiple taxa belonging to phylum *Bacteroidetes* were enriched in the model group, while RMK administration was associated with higher relative abundances of Firmicutes-related taxa including *Anaerotruncus*, *Blautia* and members of the family Lachnospiraceae. These findings indicate that tumor burden and RMK treatment were each associated with differences in the relative abundance of specific taxa, identified at an uncorrected *p* < 0.05 threshold; no false-discovery-rate correction was applied, and the results are therefore exploratory. The taxonomic shifts cannot be related quantitatively to the immunological readouts: the 16S libraries were generated from a different cohort of animals from those used for flow cytometry, the flow cytometric results are available only as group means without per-animal values, and the taxon-level analysis rests on three biological replicates per group. The relationship was examined only for directional consistency, and no correlation coefficients are reported. Because RMK also reduced tumor burden, the taxonomic differences may equally reflect a smaller tumor mass rather than a microbial mediation of tumor suppression, a possibility that the present design cannot exclude. In the remainder of this section, statements about SCFA production and about SCFA-mediated T-cell regulation are drawn from the literature and are not measurements made in this study.

At the level of the community, dietary peptides themselves can reshape microbial composition, both by supplying utilizable nitrogen and by exerting direct antimicrobial effects [35]. Beyond taxonomy, and on the basis of published work rather than of measurements made here, the taxa enriched by RMK are the principal butyrate-producing lineages of the mammalian colon: Lachnospiraceae, Ruminococcaceae and Oscillospiraceae harbor most of the known butyryl-CoA:acetate CoA-transferase and butyrate kinase routes, and *Blautia* contributes propionate and acetate through distinct pathways [36]. The concurrent contraction of Bacteroides and Prevotellaceae removes lineages whose enrichment is usually read as a marker of the inflammatory, mucin-degrading configuration that accompanies epithelial stress. Short-chain fatty acids (SCFAs) modulate host immunity by activating G-protein-coupled receptors and inhibiting histone deacetylases, which facilitates peripheral regulatory T-cell differentiation and maintains CD8^+^ T-cell effector and memory programs. Specific commensal consortia are capable of inducing CD8^+^ T-cell-dependent antitumor immunity [37,38]. SCFAs were not quantified in this study, and fecal and serum SCFA concentrations would be required to test whether the taxonomic shifts reported here translate into altered production. Collectively, these findings indicate that RMK administration was accompanied by differences in the relative abundance of specific taxa and raise the hypothesis that microbiota-derived SCFA-mediated immunoregulation may contribute to the observed phenotype; this hypothesis was not tested here and would require SCFA quantification, fecal microbiota transplantation, antibiotic depletion or germ-free conditions.

### 3.7. RMK Alters Fecal and Serum Metabolic Profiles

**Figure 7:**
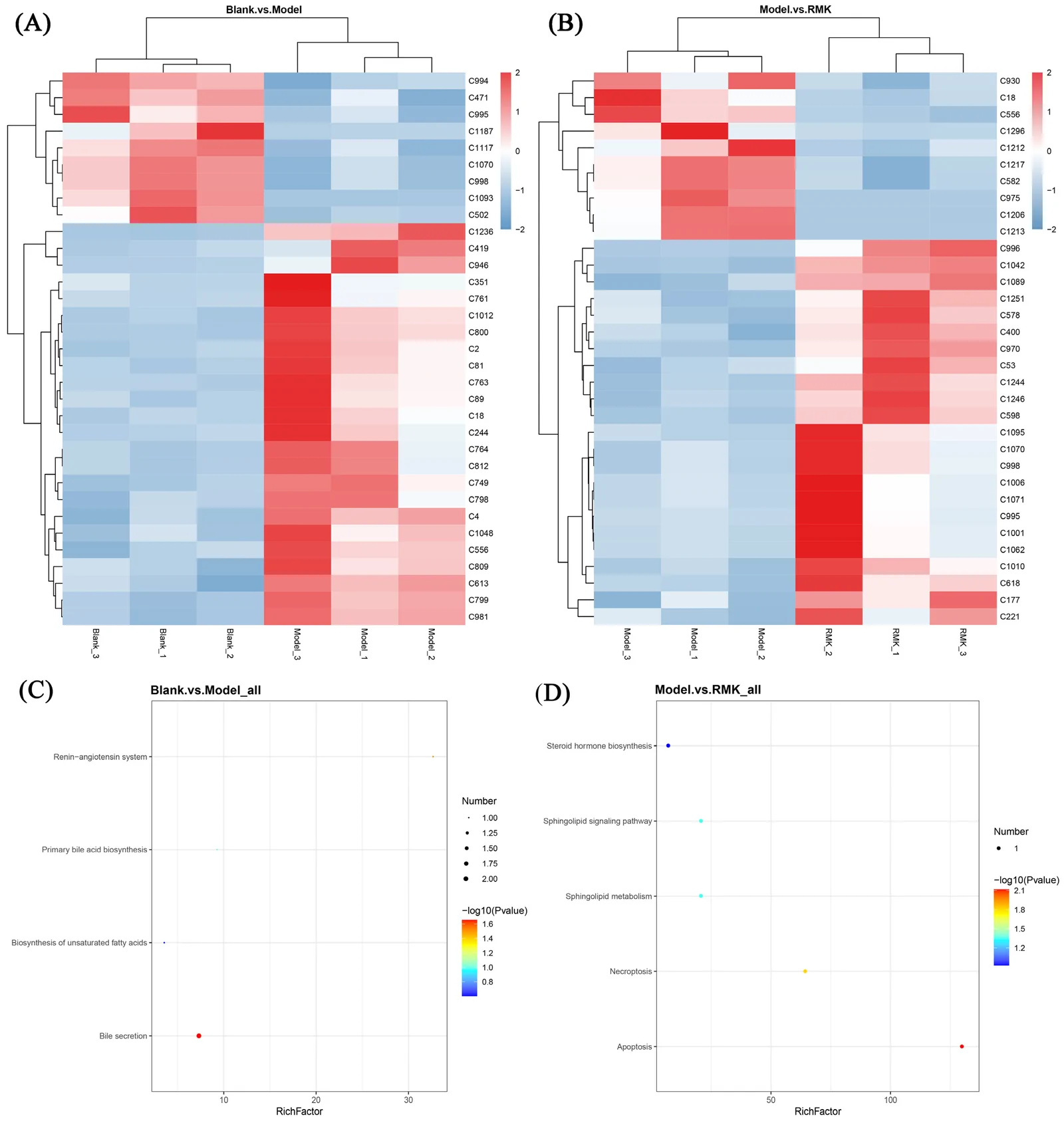
Fecal untargeted metabolomics analysis (*n* = 3). (**A**) Heatmap of differential metabolites between blank and model groups; (**B**) heatmap of differential metabolites between model and RMK groups; (**C**) KEGG pathway enrichment for blank versus model groups; (**D**) KEGG pathway enrichment for model versus RMK groups.

Figure 7 presents the fecal untargeted metabolomic analysis. The heatmap (Figure 7A) revealed obvious separation of differential metabolites between the blank and model groups, indicating that tumor growth was accompanied by altered fecal metabolic profiles. As shown in Supplemental Table S1, a total of 33 differential metabolites were identified between the two groups (*p* < 0.05), of which nine were up-regulated and 24 were down-regulated, indicating that tumor burden was associated with broad perturbation of the fecal metabolic landscape. Lipid metabolism represented the most prominent disturbed domain, as glycerophospholipids and lysophospholipids dominated the differential metabolites, including phosphatidylcholine (PC), lysophosphatidylcholine (LysoPC/LPC), lysophosphatidylethanolamine (LysoPE/LPE), phosphatidylethanolamine (PE), phosphatidylglycerol (PG) and sphingomyelin (SM). All nine LysoPC/LysoPE species were consistently down-regulated, and six fatty acid/oxylipin metabolites, including 12-HETE, 11(12)-EET, 19(20)-DiHDPA, FAHFA, 11-eicosenoic acid and an epoxy fatty acid, were also uniformly down-regulated, a pattern consistent with altered membrane phospholipid remodeling and fatty acid/oxylipin signaling. Bile acid metabolism was likewise altered, with chenodeoxycholic acid (CDCA) up-regulated while taurolithocholic acid 3-sulfate (TLCA-3-S) was markedly decreased. KEGG enrichment analysis (Figure 7C) showed that the differential metabolites were mainly enriched in bile secretion, biosynthesis of unsaturated fatty acids, primary bile acid biosynthesis and the renin–angiotensin system, further indicating that tumor growth was accompanied by altered lipid and bile acid metabolism. Collectively, these results indicate that tumor burden was accompanied by marked changes in fecal lipid, fatty acid and bile acid metabolism [39,40].

The heatmap (Figure 7B) showed a contrasting abundance pattern of numerous differential metabolites after RMK intervention, suggesting that RMK altered the metabolic phenotype of tumor-bearing mice. As shown in Supplemental Table S2, a total of 33 distinct metabolites were screened out between the model group and RMK treatment group (*p* < 0.05), of which 10 were up-regulated and 23 were down-regulated, indicating that RMK treatment was accompanied by a substantial change in the fecal metabolic profile. Glycerophospholipid remodeling was the most prominent feature: 12 of 13 PC species were significantly down-regulated after RMK intervention, partially offsetting the phospholipid changes associated with tumor growth. Concurrently, SM and sphingosine species were all up-regulated, especially SM(d18:1/24:0) (log2FC = 6.55, VIP = 5.49), so that the sphingolipid species changed in the direction opposite to that observed in tumor-bearing mice, a pattern consistent with altered sphingolipid metabolism, which is discussed below in relation to immune regulation. Moreover, steroid-hormone metabolites, including tetrahydro-11-deoxycortisol, tetrahydrocortisol and estradiol benzoate, were consistently down-regulated, and the amino acid threonine was decreased, suggesting that RMK was also associated with changes in steroid-hormone and amino acid metabolism. KEGG mapping (Figure 7D) placed apoptosis and necroptosis among the enriched pathways; given the small number of mapped metabolites, this is a pathway-level annotation rather than evidence of a death mechanism. Collectively, RMK was associated with changes in phospholipid, sphingolipid and steroid-hormone metabolites; because these are untargeted annotations without targeted confirmation, they are interpreted as metabolic associations rather than as established immunomodulatory mechanisms [41,42].

**Figure 8:**
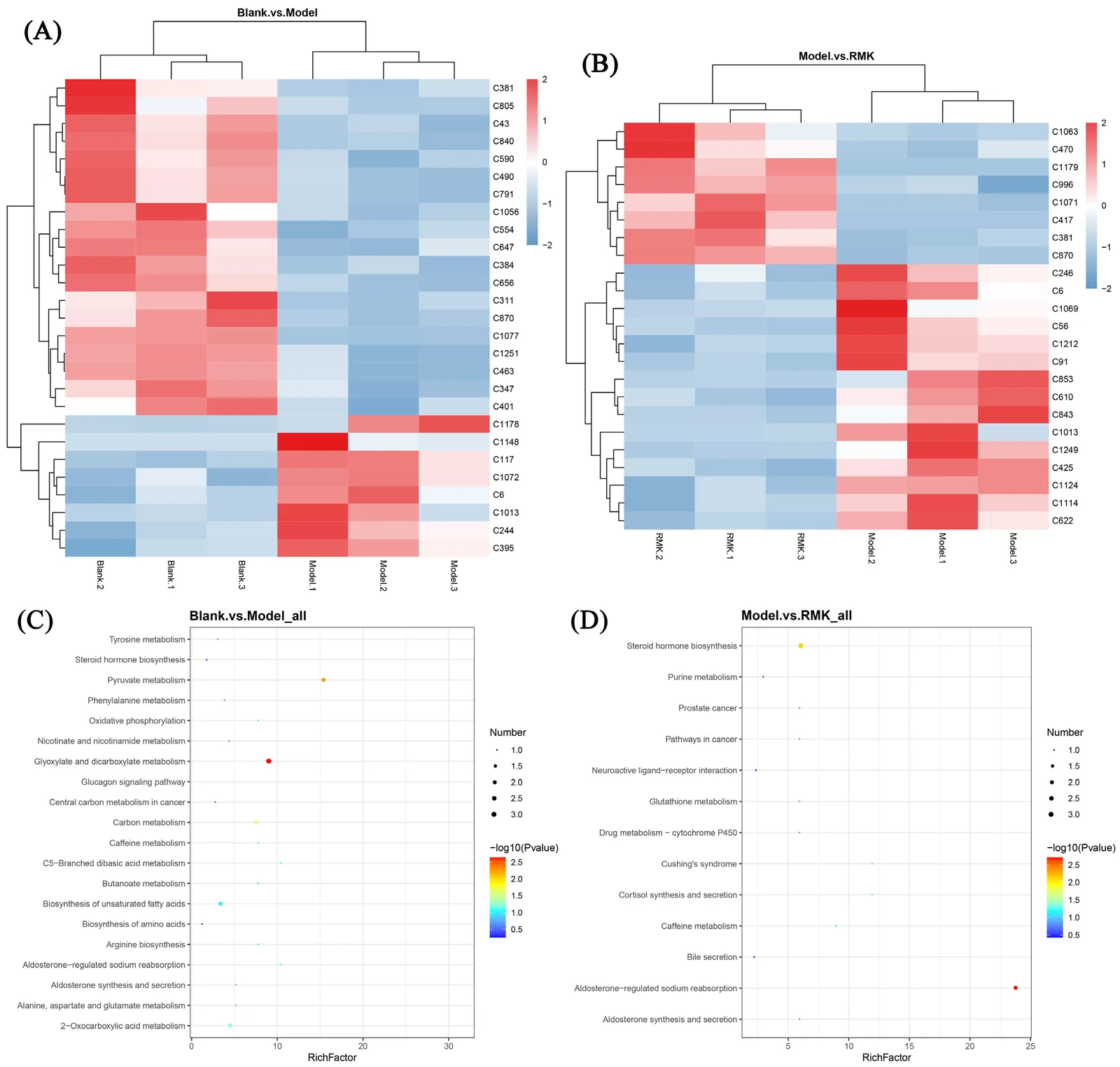
Serum untargeted metabolomics (*n* = 3). (**A**) Heatmap of differential metabolites between blank and model groups. (**B**) Heatmap of differential metabolites between model and RMK groups. (**C**) KEGG pathway enrichment for blank versus model groups. (**D**) KEGG pathway enrichment for model versus RMK groups.

Serum metabolomics identified 27 differential metabolites between the blank and model groups (19 up-regulated, eight down-regulated; Supplementary Table S3). Tumor-bearing mice exhibited marked accumulation of the phospholipid PG(16:0/16:0) and the mineralocorticoid aldosterone, together with the tricarboxylic acid cycle intermediates malate, fumarate, cis-aconitate and isocitrate, as well as eicosapentaenoic acid (EPA) and indole-related metabolites. Conversely, several sphingomyelin species—SM(d15:1/26:0) (FC = 0.02) and SM(d18:1/20:0) (FC = 0.03)—and the ether phospholipid PC(22:6e/18:4) were strongly depleted. Hierarchical clustering separated the blank and model groups (Figure 8A). KEGG enrichment identified glyoxylate and dicarboxylate metabolism as the most significantly perturbed pathway, followed by pyruvate metabolism, butanoate metabolism, tyrosine metabolism, steroid-hormone biosynthesis and oxidative phosphorylation (Figure 8C), a pattern consistent with systemic metabolic reprogramming during tumor growth [43,44].

RMK treatment altered the levels of 23 serum metabolites relative to the model group (15 up-regulated, eight down-regulated; Supplementary Table S4). RMK markedly reduced the levels of aldosterone, cortisol and tetrahydrocortisone, as well as SM(d18:1/22:0) and arachidonoyl-containing phosphatidylethanolamines PE(16:1e/22:6), PE(18:2e/20:3) and PE(22:4e/20:4). In contrast, PC(22:6e/18:4) was higher after RMK treatment, accompanied by elevated prostaglandin D3, pyroglutamic acid, guanine and caffeine. Clustering again separated the model and RMK groups (Figure 8B). KEGG analysis identified aldosterone-regulated sodium reabsorption as the most significant pathway, together with steroid-hormone biosynthesis, cortisol synthesis and secretion, glutathione metabolism, bile secretion and caffeine metabolism (Figure 8D). Circulating aldosterone and cortisol were therefore lower after RMK treatment than in tumor-bearing mice; both were annotated by untargeted profiling without targeted confirmation, and neither receptor occupancy nor glucocorticoid-responsive gene expression nor T-cell function was measured, so these are reported as metabolite-level differences and are not interpreted as relief of glucocorticoid-mediated immunosuppression. The enrichment of glutathione metabolism is likewise reported as a pathway-level observation consistent with altered redox metabolism rather than as independent evidence of mitochondrial injury [45,46]. The relationship between these metabolites and T-cell function is at present indirect: neither the steroid concentrations (targeted) nor their downstream effects on T cells were measured, and all annotations remain putative; establishing such a relationship would require targeted quantification together with functional readouts of T-cell activation in the same animals.

### 3.8. Exploratory Molecular Docking of RMK Against Candidate Immune Targets

Across 18 targets, the lowest-energy poses were numerically most favorable for molecules acting in antigen presentation and TCR-proximal signaling and least favorable for the classical checkpoint pair PD-1 and CTLA-4 (Table 1). Because a single ligand was docked against structurally unrelated proteins, and because no control peptide, replicate docking campaign or alternative scoring function was included (Section 2.9), this ranking is at most indicative and is not a measure of affinity or selectivity.

**Figure 9:**
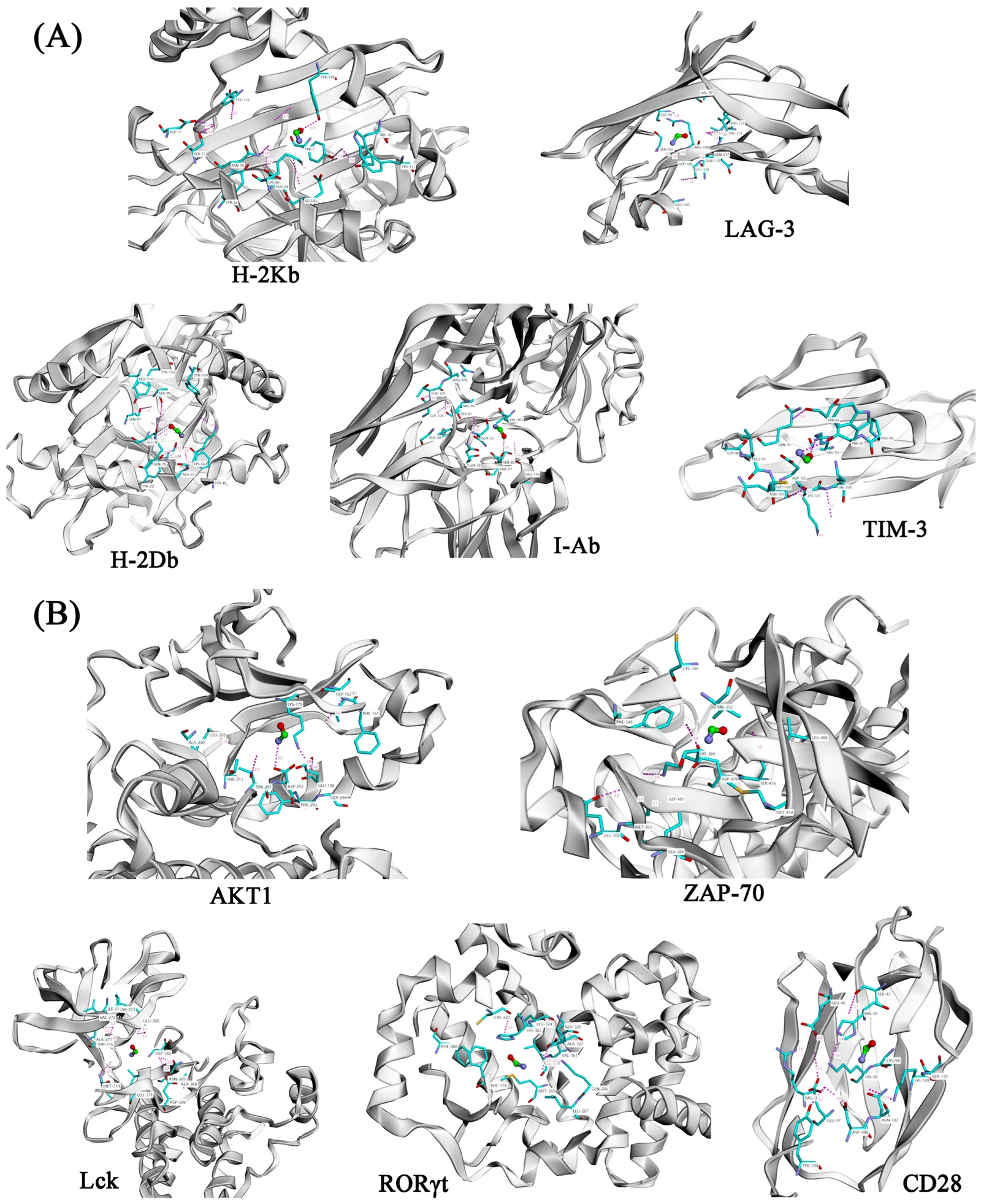
Representative docking conformations of RMK with immune-related targets. (**A**) Murine antigen-presentation and checkpoint targets: H-2Kb, H-2Db, I-Ab, LAG-3 and TIM-3. (**B**) Human TCR signaling and lineage targets: AKT1, ZAP-70, Lck, RORγt and CD28. Protein backbones are shown as cartoons, RMK as sticks, and hydrogen bonds as dashed lines.

Among murine targets, H-2Kb showed the lowest binding free energy (−7.09 kcal/mol, 21 contact residues), followed by LAG-3 (−6.67), H-2Db (−6.61) and the MHC-II molecule I-Ab (−6.31, 24 contact residues); TIM-3 (−5.05) reached the threshold for potential binding, whereas NF-κB p50 (−4.72) was classified as weak and T-bet (−3.63) showed no substantial interaction. Among human targets, AKT1 (−6.92, 21 residues), ZAP-70 (−6.70) and Lck (−6.61, 21 residues) were most favorable, followed by RORγt (−6.08), CD28 (−5.62), TIGIT (−5.18) and STAT5B (−5.14); FOXP3 (−4.93) and ICOS (−4.88) were weak, and PD-1 (−4.50) and CTLA-4 (−4.21) did not meet the criterion. The representative poses (Figure 9) place RMK within the peptide-binding grooves of H-2Kb, H-2Db and I-Ab and within surface pockets of AKT1, ZAP-70 and Lck, that is, in the groove for the MHC molecules and in pockets adjacent to the ATP-binding cleft of AKT1 and to the catalytic cleft of Lck and ZAP-70. These are geometric descriptions of the lowest-energy pose of a single docking campaign and do not indicate that such complexes form; pocket location alone does not show that a functional site has been identified.

The five murine targets with ΔG ≤ −5.0 kcal/mol spanned the antigen-presentation arm of the panel: two MHC-I alleles (H-2Kb, −7.09; H-2Db, −6.61), one MHC-II allele (I-Ab, −6.31) and two checkpoint molecules (LAG-3, −6.67; TIM-3, −5.05). H-2Kb and H-2Db are among the most widely studied classical class I restriction elements in mice, and the poses obtained for RMK placed it within their peptide-binding grooves, with 21 and 16 contact residues, respectively; the number of contact residues reflects the size of the docked pose rather than a binding affinity. A tripeptide is substantially shorter than the 8–10-residue and 13–25-residue peptides canonically accommodated by MHC-I and MHC-II grooves, so occupancy of a groove by RMK cannot be taken as evidence of altered antigen presentation, and stable peptide–MHC assembly or altered antigen recognition would each require direct experimental demonstration; non-canonical or bulged binding cannot be excluded a priori, but it is not demonstrated here [47]. The larger number of contact residues on I-Ab (24) does not indicate preferential class II engagement, and no link is drawn between this pose and the CD4^+^ T-cell proportions reported above. RMK also gave favorable scores for LAG-3 and TIM-3, both of which are up-regulated on dysfunctional intratumoral CD8^+^ T cells and are being targeted clinically to restore effector function [48,49]. These scores indicate only that the modeled structures present a surface groove geometrically able to accommodate a tripeptide; they do not constitute evidence of checkpoint inhibition.

The five human targets with the most favorable ΔG values—AKT1 (−6.92), ZAP-70 (−6.70), Lck (−6.61), RORγt (−6.08) and CD28 (−5.62)—were the numerically lowest-scoring entries of the human panel, although scores obtained for structurally unrelated proteins are not directly comparable and treating them as a group is an interpretive convenience rather than a demonstrated pathway. The functions of these molecules are established: Lck, a Src-family kinase, phosphorylates CD3 ITAMs and ZAP-70 upon TCR engagement, ZAP-70 propagates the signal to LAT and SLP-76, and AKT1, recruited downstream of CD28–PI3K co-stimulation, phosphorylates mTOR and FOXO to promote T-cell survival, glucose uptake and effector differentiation [50,51]; the −6.92 kcal/mol pose on AKT1, with 21 contact residues, was the numerically lowest of the human entries. Lck, ZAP-70 and AKT1 are intracellular targets, and no evidence is presented that RMK enters lymphocytes, so no prediction of this kind can establish simultaneous engagement within a cell. Because the biological experiment was performed in mice whereas these five receptors were modeled from human structures, computational scores obtained against human proteins cannot retrospectively explain a murine phenotype, and their bearing on the thymic DP proportions, the peripheral CD4^+^ and CD8^+^ pools and the intratumoral CD8^+^ content reported above remains untested [52]. The less favorable scores obtained for PD-1, CTLA-4, T-bet, FOXP3 and ICOS do not indicate that these molecules are not engaged in vivo.

## 4. Discussion

### 4.1. Principal Findings: Immunological, Microbial and Metabolic Correlates of the Antitumor Effect

In a host-preconditioning plus continuous-exposure design, oral administration of the tripeptide RMK reduced the growth of subcutaneously inoculated S180 sarcoma at both doses. The distinction between demonstrated findings and proposed mechanisms should be made at the outset. Demonstrated are lower tumor weight, different proportions of gated T-cell subsets in thymus, spleen, blood and tumor, different relative abundances of specific bacterial taxa, different levels of putatively annotated fecal and serum metabolites, and lowest-energy docking poses for particular proteins; not demonstrated are increased thymocyte production, T-cell activation, tumor reactivity or cytotoxic killing, recruitment or redistribution of lymphocytes, short-chain fatty acid-mediated immunoregulation, target occupancy by RMK, a specific mode of cell death, or activity against an established tumor. Because every immunological readout is a proportion of gated cells, a changed subset distribution cannot be read as production, activation, killing or recruitment; an increased intratumoral CD8^+^ proportion is a prerequisite for effective tumor immunotherapy but is not a measure of it [53]. Peptide therapeutics occupy a unique niche in this field, as they can be engineered to act at multiple steps of the cancer-immunity cycle [54]. Consistent with this, our findings suggest that RMK acts upstream at the gut-immune interface and is associated with immunological changes downstream of that interface. Nevertheless, the current experimental design cannot rule out the alternative explanation that reduced tumor burden itself partly accounts for these differences: tumor burden alters gut ecology and systemic metabolism, and because dosing began seven days before inoculation, the study cannot separate an effect on tumor establishment from an effect on the growth of an already established tumor.

### 4.2. The Gut Microbiota–Metabolite Axis as a Working Hypothesis

The microbiome and metabolome data describe the compartment most directly exposed to orally administered RMK and are therefore the most plausible link to its systemic effects, although they do not establish that link. With three biological replicates per group, the relative abundances of Bacteroides and Prevotella were lower and those of Lachnospiraceae, Ruminococcaceae, Oscillospiraceae and Anaerotruncus higher after RMK treatment, whereas community-level structure did not differ significantly between the model and RMK groups and alpha diversity separated the groups poorly; these taxa include major colonic short-chain fatty acid (SCFA)-producing lineages [55]. Interpretation is correspondingly narrow: SCFAs have been reported to enhance CD8^+^ T-cell effector functions and memory differentiation and to improve responses to checkpoint blockade and adoptive cell therapy, and in the studies cited, microbiota composition behaves as a causal determinant rather than a passive correlate of CD8^+^ T-cell-dependent antitumor immunity [56], whereas the present dataset does not allow a comparable inference; the directional consistency among microbiome, metabolome and immunological readouts reported here aligns with that literature but remains correlative, and because tumor burden itself alters gut ecology, these differences cannot be separated from the effect of the smaller tumor in RMK-treated animals. Testing the relationship would require a paired design in which taxa, SCFA concentrations and T-cell readouts are measured in the same animals, together with fecal microbiota transplantation, antibiotic depletion or germ-free recipients, and quantification of bile acids, sphingolipids and steroids; none of these approaches were used here.

### 4.3. Candidate Molecular Targets: An Explicitly Speculative Model

Molecular docking served here only as a hypothesis-generating exercise: it cannot establish binding, target occupancy or functional modulation, and none of the predictions below are supported by biochemical or signaling data in this study. With that qualification, the lowest-energy poses for RMK were obtained for MHC class I and class II molecules, the TCR-proximal kinases Lck and ZAP-70, AKT1 and the co-stimulatory receptor CD28, rather than for the classical checkpoint pair. Computational docking is widely used to model peptide–MHC and TCR–pMHC interactions and to rank ligand poses before experimental validation [57], and peptide conformation within the MHC groove has been reported to modulate TCR triggering strength and the recruitment of proximal signaling kinases [58]. Whether intact RMK reaches any of these targets is unresolved. Intact tripeptides are susceptible to brush-border and plasma peptidases, and although PepT1 transports di- and tripeptides across the enterocyte, transport is not equivalent to systemic exposure of the intact peptide, nor to its uptake by lymphocytes. In an earlier, unpublished, semi-quantitative experiment from our group, oral administration of synthetic RMK did not noticeably raise the plasma level of the intact tripeptide; the assay was not a validated quantitative method, and the observation is reported here only qualitatively, without numerical values. Read together, these considerations argue that the principal site of RMK action is more likely to be the intestinal lumen and the associated microbiota than the systemic circulation, which makes the microbial and metabolic pathway discussed in Section 4.2 the more plausible main line of interpretation and relegates the docking output to a secondary, exploratory role. Accordingly, predictions involving the intracellular kinases Lck, ZAP-70 and AKT1 are best regarded as speculative, and the compartments in which RMK acts remain to be defined by quantitative pharmacokinetic and biodistribution studies.

### 4.4. Limitations

Several limitations qualify these conclusions. First, no positive comparator was included, so the magnitude of the effect cannot be benchmarked against established agents. Second, and most important for translation, RMK was given from 7 days before to 14 days after inoculation with no treatment-only arm, so the design cannot separate an effect on tumor establishment from an effect on the growth of an established tumor. Third, all flow cytometric readouts are proportions of gated cells, and absolute counts and organ cellularity were not obtained, which constrains interpretation of the thymic data in particular. Fourth, no assay of T-cell effector function (IFN-γ, granzyme B, perforin, degranulation or CD8 depletion) was performed. Fifth, the microbiome–metabolite–immune relationship remains associative: three replicates per group, uncorrected taxon-level tests, a non-significant PERMANOVA and no SCFA quantification; fecal transplantation and antibiotic depletion, the approaches that would permit a causal test [59,60], were not performed. Sixth, metabolites were putatively annotated, without authentic standards, false-discovery-rate correction or targeted validation.

## 5. Conclusions

The synthetic immunoactive tripeptide RMK, delivered orally in a preconditioning and continuous-exposure scheme before and after tumor challenge, inhibited S180 tumor growth at both doses. RMK was associated with increased thymic CD4^+^CD8^+^ DP cells, splenic and intratumoral CD4^+^/CD8^+^ T cells, which cannot directly prove tumor reactivity or cytotoxicity. It triggered mitochondrial depolarization and ROS accumulation in tumor suspensions, enriched SCFA-producing Firmicutes (Lachnospiraceae, Ruminococcaceae) (SCFAs unmeasured), and modulated bile acid, sphingolipid and steroid-hormone metabolism with decreased aldosterone and cortisol. Docking showed more favorable poses toward MHC-I/II, Lck, ZAP-70 and AKT1 than PD-1/CTLA-4; such computational predictions are speculative without pharmacokinetic data. These results inform future validation assays: LC-MS/MS pharmacokinetics, SCFA and targeted metabolite detection, CD8 depletion, FMT and biochemical binding tests for the proposed model.

## Figures and Tables

**Table 1:** Molecular docking results for RMK against 18 immune-related targets.

Rank	Gene	Protein	PDB	Category	ΔG (kcal/mol)	Contact Residues	Predicted Binding
Murine targets							
1	H2-K1	H-2Kb	1VAC	MHC-I antigen presentation	−7.09	21	Potential
2	Lag3	LAG-3	7TZE	Immune checkpoint	−6.67	18	Potential
3	H2-D1	H-2Db	1HOC	MHC-I antigen presentation	−6.61	16	Potential
4	H2-Ab1	I-Ab	6MNG	MHC-II antigen presentation	−6.31	24	Potential
5	Timd3	TIM-3	3KAA	Immune checkpoint	−5.05	12	Potential
6	Nfkb1	NF-κB p50	1NFK	Transcription factor	−4.72	11	Weak
7	Tbx21	T-bet	5T1J	Lineage transcription factor	−3.63	10	No substantial binding
Human targets							
1	Akt1	AKT1	3CQW	Downstream signaling kinase	−6.92	21	Potential
2	Zap70	ZAP-70	1U59	TCR signaling kinase	−6.70	15	Potential
3	Lck	Lck	3LCK	TCR signaling kinase	−6.61	21	Potential
4	Rorc	RORγt	3L0L	Lineage transcription factor	−6.08	17	Potential
5	Cd28	CD28	1YJD	Co-stimulatory receptor	−5.62	15	Potential
6	Tigit	TIGIT	3UDW	Immune checkpoint	−5.18	13	Potential
7	Stat5b	STAT5B	6MBW	Transcription factor	−5.14	18	Potential
8	Foxp3	FOXP3	3QRF	Lineage transcription factor	−4.93	9	Weak
9	Icos	ICOS	6X4G	Co-stimulatory receptor	−4.88	12	Weak
10	Pdcd1	PD-1	3RRQ	Immune checkpoint	−4.50	11	No substantial binding
11	Ctla4	CTLA-4	3OSK	Immune checkpoint	−4.21	9	No substantial binding

Binding was classified as potential (ΔG ≤ −5.0 kcal/mol), weak (−5.0 to −4.5 kcal/mol) or no substantial binding (>−4.5 kcal/mol). This cut-off is an operational reporting convention and not a validated affinity criterion: scores obtained for proteins that differ in size and in pocket hydrophobicity cannot be compared with one another, and the categories serve only to rank poses within this exploratory exercise. Contact residues are those within 4 Å of the docked RMK conformation; they describe geometric proximity alone and are not a measure of interaction energy or of binding affinity.

## Data Availability

All data supporting the findings of this study are available from the corresponding authors upon reasonable request.

## Supplementary Materials

The following supporting information can be downloaded at: https://www.mdpi.com/article/10.3390/nu18213628/s1. Supplementary Table S1: Differential fecal metabolites between blank and model groups; Supplementary Table S2: Differential fecal metabolites between model and RMK groups; Supplementary Table S3: Differential serum metabolites between blank and model groups; Supplementary Table S4: Differential serum metabolites between model and RMK groups.
